# Pulmonary Carcinoid Tumors (Lung Carcinoids): Current Concepts in Diagnosis and Management

**DOI:** 10.3390/ijms27198868

**Published:** 2026-10-05

**Authors:** David L. Chan, Yuji Uehara, Keigo Kobayashi, Aaron C. Tan

**Affiliations:** 1 Northern Clinical School, University of Sydney, Camperdown, Sydney 2050, Australia; 2 Department of Medical Oncology, Royal North Shore Hospital, Sydney 2065, Australia; 3 Department of Experimental Therapeutics, National Cancer Center Hospital, Tokyo 104-0045, Japan; 4 Department of Internal Medicine, Japan Green Vietnam Clinic, Hanoi 11908, Vietnam; 5 Division of Medical Oncology, National Cancer Centre Singapore, 30 Hospital Boulevard, Singapore 168583, Singapore; 6 Duke-NUS Medical School, National University of Singapore, Singapore 169857, Singapore

**Keywords:** lung carcinoid, neuroendocrine neoplasm, pulmonary carcinoid

## Abstract

Pulmonary carcinoid tumors are rare lung neuroendocrine neoplasms with diverse clinical behavior, ranging from indolent localized disease to metastatic progression. Diagnosis relies on histopathology but may be supplemented by immunohistochemical markers such as Ki-67. Recent molecular and multi-omic studies reveal substantial heterogeneity, identifying molecularly defined subgroups and highly proliferative carcinoids with aggressive behavior. Management includes surgical resection for localized disease and systemic therapies for advanced cases, such as somatostatin analogs, targeted agents, peptide receptor radionuclide therapy, and chemotherapy. While emerging molecular classifications and biomarker-driven strategies hold significant promise, current management of advanced pulmonary carcinoids relies heavily on extrapolated data from mixed neuroendocrine cohorts, underscoring the urgent need for prospective thoracic-dedicated trials to validate these paradigms in clinical practice. Emerging biomarker-driven trial designs and integrated precision medicine approaches offer opportunities to optimize therapy and improve outcomes in this rare tumor type.

## 1. Introduction

Pulmonary carcinoid tumors are rare lung neuroendocrine neoplasms whose clinical and biological diversity has long challenged diagnosis and management. Emerging molecular and immunophenotypic insights are now revealing distinct subtypes with prognostic and therapeutic relevance, highlighting opportunities for more precise, personalized interventions. Yet, the scarcity of cases and conventional trial designs has limited evidence generation, underscoring an urgent need for innovative strategies to translate these discoveries into meaningful patient outcomes. The classification is also evolving. The 2021 WHO thoracic classification continues to separate well-differentiated pulmonary carcinoids (typical and atypical carcinoids) from poorly differentiated neuroendocrine carcinomas (small-cell lung carcinoma and large-cell neuroendocrine carcinoma), whereas the broader WHO/IARC neuroendocrine neoplasm framework distinguishes well-differentiated neuroendocrine tumors from poorly differentiated neuroendocrine carcinomas across organs [1,2,3]. In this context, highly proliferative pulmonary carcinoids, or bronchopulmonary NET G3-like tumors, remain an emerging and debated category: they retain carcinoid/NET morphology but show mitotic or Ki-67 activity above conventional atypical carcinoid thresholds [4,5]. Integrating morphology, proliferation indices, immunophenotype, and molecular data is therefore essential for refining classification and linking biological subtypes to management.

The literature for this narrative review was synthesized from a comprehensive range of relevant resources (PubMed/MEDLINE), including peer-reviewed prospective clinical trials, pivotal retrospective series, international expert guidelines (ENETS, NANETS, ESMO, and NCCN), multi-omic classification frameworks, and recent major conference proceedings.

## 2. Pathology and Molecular Biology

### 2.1. Overview of WHO Classification

**Figure 1:**
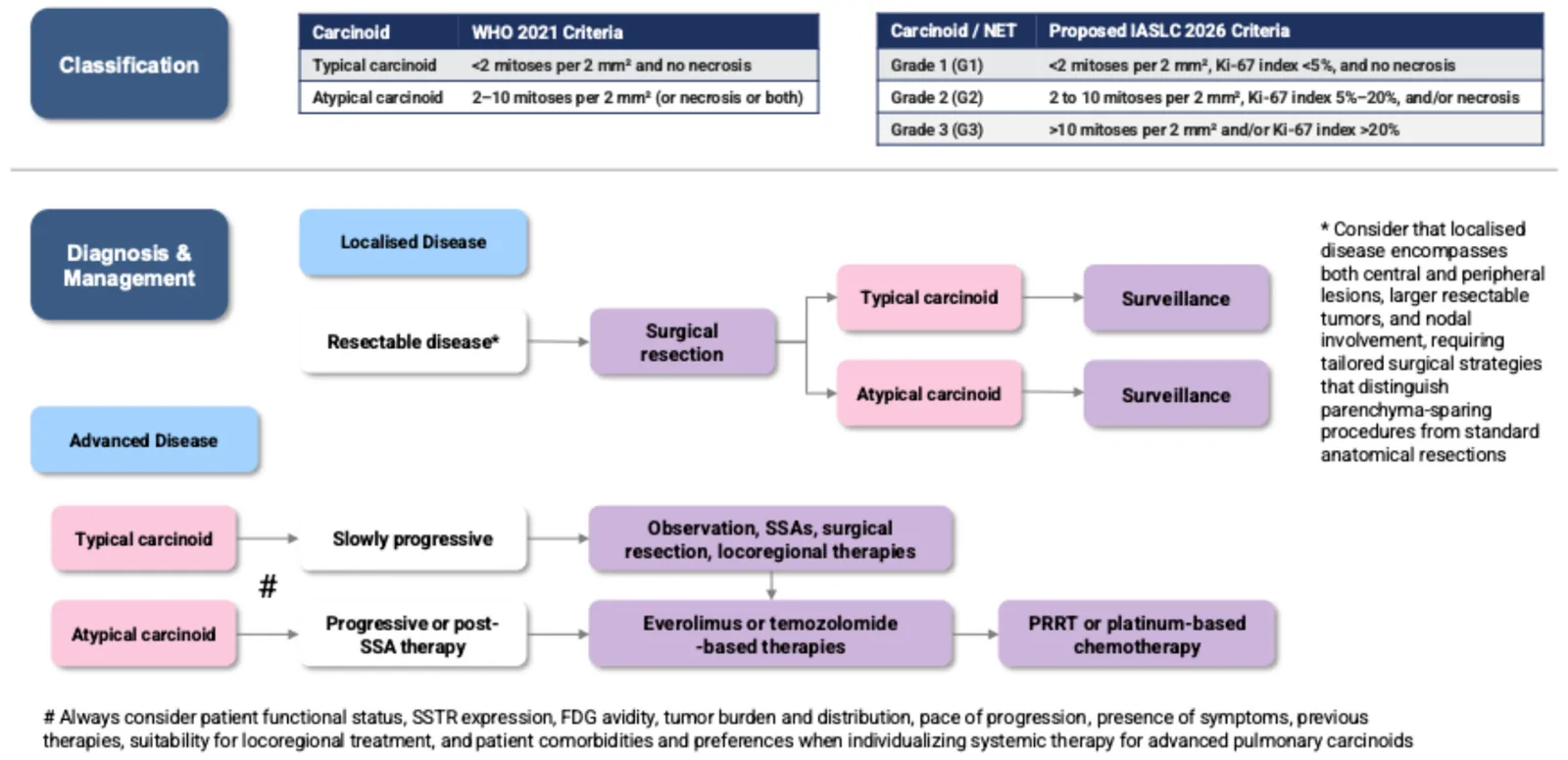
Pulmonary carcinoid classification, diagnosis, and management.

The classification of pulmonary neuroendocrine neoplasms (NENs) continues to evolve. Currently, the 2021 World Health Organization (WHO) thoracic schema classifies NENs into well-differentiated pulmonary carcinoids, comprising typical carcinoid (TC) and atypical carcinoid (AC), and poorly differentiated neuroendocrine carcinomas (NEC), comprising small-cell lung carcinoma (SCLC) and large-cell neuroendocrine carcinoma (LCNEC) [1,2,3]. This classification is primarily based on morphology, mitotic count, and the presence or absence of necrosis (usually focal/punctate or comedo-like) (Figure 1). TC is defined as having fewer than two mitoses per 2 mm^2^ and no necrosis. AC has a mitotic rate of 2 to 10 mitoses per 2 mm^2^ or exhibits necrosis. Tumors with unequivocal NEC morphology are classified as SCLC or LCNEC, but rare tumors with carcinoid morphology and proliferation above conventional AC thresholds have been described as highly proliferative carcinoids or bronchopulmonary NET G3-like tumors [4,5]. These tumors are not yet a formal WHO thoracic category, but they underscore that morphology, proliferation, and molecular features should be interpreted together rather than using mitotic count or Ki-67 alone.

Most recently, however, a 2026 position paper from the International Association for the Study of Lung Cancer (IASLC) has proposed a transition toward a broader classification consistent with other neuroendocrine tumors [6]. This classification consists of carcinoid/NET G1, G2, and G3 (Figure 1). Crucially, Ki-67 would now be incorporated as a formal diagnostic criterion alongside mitotic count and necrosis and carcinoid/NET G3 would be formally recognized as a rare, highly proliferative subset that retains well-differentiated NET architecture and molecular traits rather than high-grade NEC features.

In addition, there is the entity of diffuse idiopathic pulmonary neuroendocrine cell hyperplasia (DIPNECH). DIPNECH is a rare, preinvasive proliferation of pulmonary neuroendocrine cells in the bronchial epithelium that can lead to airway inflammation, constrictive bronchiolitis, and the formation of tumorlets (<5 mm) or carcinoid tumors (≥5 mm) [7]. Serving as the primary preinvasive precursor lesion in the spectrum of pulmonary NENs, DIPNECH can coexist with or give rise to multifocal low-grade carcinoid tumors.

### 2.2. The Role of Immunohistochemistry 

The current WHO classification of lung carcinoids primarily uses morphological characteristics to differentiate TC and AC [1]. However, relying solely on morphology may not always ensure accurate diagnosis, particularly in challenging cases such as small biopsies where crush artifacts can resemble more aggressive tumors. Ki-67 is useful as an adjunct, analogous to its established role in gastrointestinal and pancreatobiliary neuroendocrine tumors, but it is not mandatory for classifying pulmonary carcinoids into TC and AC [1,2]. IHC for Ki-67 can be instrumental in distinguishing crush artefact in carcinoids from SCLC in biopsies, a frequent diagnostic challenge, as the Ki-67 index is generally low in carcinoids (1–20%, often <10%) and high in NEC (>50%) [8,9]. In the emerging highly proliferative carcinoid/bronchopulmonary NET G3 context, Ki-67 and mitotic count should be interpreted with carcinoid morphology and p53/RB status rather than used in isolation because these tumors can retain well-differentiated architecture and often lack the TP53/RB1 alterations typical of NECs [4,5]. Under the 2026 IASLC update, Ki-67 has been formally incorporated as a diagnostic criterion alongside mitotic count and necrosis, rather than functioning merely as an optional adjunct [6]. 

Carcinoids generally exhibit diffuse positivity for all standard NE markers (synaptophysin, chromogranin A, CD56) and a newer NE marker (INSM1), although INSM1 can also be present in other NE tumors [10,11,12]. TTF-1 is positive in some carcinoids (30–50%) and may support, but does not establish, pulmonary origin in metastatic cases because its expression is not exclusive to carcinoids or pulmonary origin. IHC for orthopedia homeobox protein (OTP), which is highly specific for pulmonary carcinoids and generally absent from NEC, NSCLC, and well-differentiated NETs of non-pulmonary origin, is considered potentially useful for identifying metastatic carcinoids of lung origin, though the actual prevalence of OTP-positive carcinoids in metastases is uncertain and requires clinical validation [2,13,14,15,16]. However, its routine clinical integration is currently limited by restricted laboratory availability and a lack of standardized commercial staining platforms. IHC for DLL3 is positive in a lung carcinoid subtype with high dendritic cell infiltration and strong immune activity [15], which could have therapeutic implications given the FDA’s accelerated approval of tarlatamab for extensive-stage SCLC [17]. However, DLL3 expression remains an investigational biomarker in pulmonary carcinoids. While morphology remains the cornerstone for classification, further research into incorporating Ki-67 and other IHC markers is necessary to enhance diagnosis, prognosis, and treatment strategies for lung carcinoid tumors.

### 2.3. Histological Features Integrated with Genomic Data: Emerging Paradigm Based on Genomic & Transcriptomic Profiling

Lung carcinoids typically have a low mutational burden (0.4 mutations/Mb) and only a few recurrently mutated genes [18]. The gene most frequently mutated in carcinoids is *MEN1* (11–36%), which is involved in chromatin remodeling and often associated with loss of heterozygosity [18,19,20,21,22]. Many other genes mutated in lung carcinoids are also involved in chromatin remodeling (35–40%), which regulates gene expression by altering the chromatin structure to either permit or block the binding of transcriptional regulatory proteins to DNA [15,18,19,20]. These genes can be categorized into covalent histone-modifying complexes (involving acetylation, methylation, phosphorylation, and ubiquitination: e.g., *MEN1*, *SETD1B*, *KMT2A (MLL)*), and ATP-dependent chromatin remodeling complexes (such as the SWI/SNF complex: e.g., *ARID1A*, *SMARCA4*, *SMARCB1*) [13]. Mutations in tumor suppressor genes like *TP53*, *RB1*, and *ATM* are rare, occurring in less than 5% of cases [15,19,20]. Exome sequencing analysis has identified mutations in the MAPK/ERK and amyloid-beta precursor protein pathways in lung carcinoids [23]. Additionally, germline mutations in *MEN1* are found in 2% of patients with lung carcinoid and are typically associated with multiple endocrine neoplasia type 1 syndrome [24]. 

Recent multi-omic studies suggest that lung carcinoids consist of four or three distinct biological subtypes based on gene expression, methylation, and mutation profiles (A1/A2/B/supra-carcinoid or LC1/LC2/LC3) [15,19]. These subtypes do not strictly correlate with the traditional classifications of typical and atypical carcinoids but show significant associations with tumor location (central vs. peripheral), age (younger vs. older), and prognosis [19]. For instance, one subtype characterized by *MEN1* mutations and low OTP expression has been linked to poorer outcomes, highlighting the limitations of relying solely on histological features for prognostication [15,19]. 

Translating these genomic classes into routine practice is becoming more feasible, although molecular testing is not yet a mandatory standard of care in broad clinical practice. An OTP/ASCL1/HNF1A immunohistochemistry panel separated resected pulmonary carcinoids into A1 (OTP-high/ASCL1-high/HNF1A-low), A2 (OTP-high/ASCL1-low/HNF1A-high), and B (OTP-low/ASCL1-low/HNF1A-high) groups [25]. These groups showed distinct clinicopathologic and potential therapeutic patterns: A1 was enriched for older female patients and peripheral tumors with lower SSTR2A and higher DLL3 expression, A2 for younger patients and central tumors with higher SSTR2A and lower DLL3 expression, and B for more male patients and more frequent recurrence [25]. Although prospective validation is required, these data link molecular subtype to practical questions such as SSTR-targeted imaging and therapy, DLL3-directed strategies, and recurrence risk [3,25]. This is particularly important with the emergence of DLL3-targeting agents with proven efficacy in small-cell lung cancer [26,27]. 

Supra-carcinoids should be discussed as a multi-omic, LCNEC-like aggressive pulmonary carcinoid subset rather than being equated directly with G3 NET. In the lungNENomics analyses, supra-carcinoids retained carcinoid-like morphology in some cases but showed molecular and clinical features closer to LCNEC, with a reported frequency of approximately 6% and poor survival, similar to LCNEC [15,28,29]. This concept overlaps conceptually with highly proliferative/G3 carcinoid discussions but is not identical: highly proliferative carcinoids are defined pathologically by carcinoid/NET morphology with proliferation above conventional AC thresholds, whereas supra-carcinoids are defined by integrated molecular profiles [4,5]. Some supra-carcinoids also exhibit neutrophil infiltration and immune checkpoint gene expression, raising the hypothesis that immune-based or other biomarker-directed strategies may be relevant in carefully selected aggressive subsets [15,29]. Together, these findings support refinement of lung NEN classification by integrating histology, proliferation, immunophenotype, and multi-omic data [3]. 

A separate, emerging concept relevant to the carcinoid spectrum is atypical SCLC (aSCLC), a rare clinically aggressive SCLC subset lacking the usual concurrent RB1 and TP53 inactivation and arising disproportionately in never/light smokers [30]. Chromothripsis with extrachromosomal DNA (ecDNA) amplification of CCND1 or co-amplification of CCND2/CDK4/MDM2 appears central to its biology, and pathologic and genomic links to pulmonary carcinoids suggest possible transformation from lower-grade neuroendocrine tumors or their progenitors [30]. Despite this histogenetic link, aSCLC remains a high-grade SCLC entity clinically, and its recognition underscores the broader lung NEN spectrum and the need to distinguish lineage, differentiation, and proliferative grade.

## 3. Clinical Presentation

Pulmonary carcinoids present with symptoms similar to other lung tumors. Some patients are diagnosed after investigation for cough, wheeze, hemoptysis, or infection (related to bronchial obstruction from the primary). Yet others are incidentally diagnosed on imaging for other reasons (e.g., CT coronary angiogram), and a significant minority present with symptoms of metastatic disease, whether that be related to an organ (e.g., abdominal discomfort from hepatic infiltration) or systemic symptoms of fatigue, weight loss, and/or night sweats related to tumor bulk [31]. Bronchoscopy and biopsy may be helpful in establishing the diagnosis for centrally located pulmonary carcinoids.

In comparison to midgut carcinoids, pulmonary carcinoids are less likely to be functional (i.e., less likely to overproduce vasoactive hormones). Functional disease (carcinoid syndrome) occurred in 8% of all patients with pulmonary carcinoids in an analysis of the SEER database [32]. The most commonly secreted hormones include serotonin (diarrhea, nausea, blood pressure changes, carcinoid heart disease) and neurokinin A (flushing). Pulmonary carcinoids may also rarely secrete ACTH, resulting in Cushing’s syndrome. 

Pulmonary carcinoids are staged using the tumor-node-metastasis (TNM) system [33]. As noted above, patients may present with metastatic disease at diagnosis, and for those with resectable disease, radiological enlargement of lymph nodes may not necessarily indicate node positivity. Whilst adjuvant therapy is not routinely recommended for resected TC/AC, staging may help clinicians judge and discuss recurrence risk and personalize a follow-up strategy.

Cross-sectional imaging, mainly with computed tomography (CT), is the mainstay of imaging for pulmonary carcinoids [34]. Mediastinal or hilar lymphadenopathy may be an inflammatory reaction rather than necessarily reflecting disease spread [35]. MRI of the liver may be of value in delineating the number and extent of liver lesions, particularly in the setting of oligometastatic disease. Positron Emission Tomography/CT (PET/CT) imaging is increasingly utilized for diagnosis and staging in pulmonary carcinoids. It is well-known that somatostatin receptor (SSTR) PET, such as ^68^Ga-DOTA-octreotate, is superior to CT for staging of GEPNENs [36]; however, the expression of somatostatin receptors on pulmonary carcinoids is more heterogeneous and, thus, PET imaging is not necessarily superior to CT for pulmonary carcinoids. Whilst SSTR PET may help detect metastatic disease, its additional value over CT in small primary pulmonary carcinoids may be limited [37]. 

The ENETs guidelines recommend SSTR PET as the initial PET of choice, followed by FDG PET if SSTR PET is negative [38]. Dual PET imaging with SSTR and FDG PET is an emerging modality for imaging pulmonary carcinoids, particularly for initial staging or as part of workup for peptide receptor radionuclide therapy (PRRT). The presence of FDG-avid, SSTR non-avid (“discordant”) lesions has been shown to be a poor prognostic factor in both GEPNETs and pulmonary carcinoids [39]. However, the biological importance of these findings remains incompletely determined. Whilst the paradigm for dual PET imaging in GEPNETs indicates that FDG+SSTR- phenotypes predict highly aggressive behavior, selected patients with this imaging phenotype may have atypical carcinoids, with the intermediate course generally predicted by this histological diagnosis. Additionally, rare patients will have disease that is neither avid on FDG nor SSTR PET [40]. It remains clear that patients without significant SSTR avidity (for example, greater than liver background uptake) should not be offered PRRT treatment (see systemic treatment below). Patients who have a change in their PET phenotype, rapid growth on serial imaging, or behavior out of keeping with biopsy grade may benefit from a repeat biopsy, as a higher Ki-67 index and mitotic rate may argue for treatment of higher-grade disease.

In summary, the practical diagnostic and staging pathway integrates contrast-enhanced CT and bronchoscopic tissue acquisition with rigorous pathological review. Staging relies on anatomical nodal evaluation complemented by SSTR PET for SSTR-avid disease, with FDG PET or dual-tracer imaging reserved for atypical, high-Ki-67, or functionally discordant lesions to capture higher-grade transformation. 

## 4. Management

### 4.1. Surgical Resection

Surgical resection is the mainstay of treatment for localized pulmonary carcinoids. Surgical resection of localized pulmonary carcinoids requires tailored anatomical planning based on tumor location, distinguishing central lesions—which are optimal candidates for parenchyma, sparing sleeve, or bronchoplastic resections—from peripheral lesions suitable for standard lobectomy or select sublobar resection. Complete hilar and mediastinal lymph node evaluation remains essential across all surgical approaches to ensure accurate staging given the distinct nodal metastatic risks of typical versus atypical histology. Whilst small, asymptomatic pulmonary carcinoids may be potentially observed in very frail patients, the metastatic potential of such pulmonary carcinoids generally argues in favor of resection rather than observation in patients fit for surgery. Typical carcinoids are especially associated with an excellent prognosis post-resection, with a 98% 5-year survival rate for localized disease [41]. Whilst atypical carcinoids can behave more aggressively, they still compare favorably to classic non-small-cell lung cancers from a prognostic point of view. Complete surgical resection with systematic lymph node evaluation remains the standard curative approach for localized pulmonary carcinoid tumors, with parenchyma-sparing techniques like sleeve resection options to maximize functional lung preservation. In addition, minimally invasive approaches may offer equivalent oncological outcomes and endobronchial procedures provide useful palliative options.

Adjuvant therapy is not commonly offered in patients with typical or atypical carcinoids, as systemic treatment post-resection has not been shown to improve survival outcomes. In rare, highly selected cases of resected N2 atypical carcinoid or high-risk disease, adjuvant chemotherapy is occasionally considered on an individualized, case-by-case basis, but standard practice favors observation following complete R0 resection [38]. In contrast, those with large-cell neuroendocrine carcinomas benefit from adjuvant cisplatin-containing chemotherapy [42,43]. There is no consensus regarding the optimal follow-up strategy for patients with resected pulmonary carcinoids, although the low recurrence rate in typical pulmonary carcinoids lowers the anticipated value of routine cross-sectional imaging [44]. For example, a typical carcinoid, T1N0R0, would be followed up according to ENETs guidelines with imaging yearly for two years, followed by imaging every 3 years for 10 years. In contrast, a recent Danish multicenter study advises against any follow-up in such a setting [45]. Regular imaging may play more of a role in patients with tumor size > 3 cm, multifocal pulmonary carcinoids, or node positivity, for the duration of at least 10 years, appreciating the delayed recurrence pattern of these pulmonary carcinoids [37]. The use of somatostatin-based ^68^Ga PET/CT for surveillance should be individualized based on histopathological risk factors for recurrence, avidity on preoperative imaging where available, and availability/cost of PET imaging in the local health system. In summary, the postoperative surveillance strategy should be individualized by systematically accounting for key prognostic risk factors, including typical versus atypical histology, tumor size, nodal status, resection margin status, presence of multifocal disease or underlying DIPNECH, baseline recurrence risk, and preoperative SSTR ^68^Ga PET/CT avidity.

### 4.2. Systemic Therapies

#### 4.2.1. Somatostatin Analogs

Somatostatin analogs (octreotide, lanreotide) can be used in pulmonary NETs for both antiproliferative effects and to decrease hormonal secretion, or either alone. Whilst the PROMID and CLARINET trials established that SSAs prolonged progression-free survival in gastroenteropancreatic NETs [46,47], these trials did not include patients with carcinoids of bronchial origin. There is no definitive or adequately powered randomized evidence to date for the use of somatostatin analogs (octreotide, lanreotide) in bronchial NETs. The SPINET trial (Table 1) randomized patients with bronchial NETs to lanreotide or placebo, but was ceased early due to insufficient accrual. Comparison of the 77 patients accrued did not show significant PFS prolongation with lanreotide (16.6 mo vs. 13.6 mo, HR 0.86, 95% CI 0.48–1.95, *p* = 0.87) [48]. However, given the similarities between bronchial and GEPNETs, SSAs may be used where available for antiproliferative effects in typical and atypical carcinoids. SSAs also play an important role in the management of functional bronchial NETs. Functionality can be demonstrated by the persistence of diarrhoea and/or flushing, improvement on short-term or long-term SSAs, and elevated urinary 5-hydroxyindolacetic (5-HIAA) assays. Patients with SSTR-negative lung carcinoids generally should not receive SSA therapy given the lack of receptor expression. However, the exception is for the minority of patients who present with functional, hormone-secreting tumors; SSAs remain an important therapeutic option for anti-secretory symptom control regardless of SSTR ^68^Ga PET radiotracer avidity. Common side effects of SSAs include self-limiting gastrointestinal upset and diarrhea and cholelithiasis with long-term use.

#### 4.2.2. Targeted Therapies

Targeted agents, treatments targeted at pathways of bronchial NET growth, play a significant part in systemic treatment. Whilst targeted agents may be associated with more side effects than SSAs, there is robust randomized data attesting to their antiproliferative efficacy. Everolimus is an oral inhibitor of the mammalian target of rapamycin (mTOR) pathway. The RADIANT-4 trial showed that everolimus prolonged progression-free survival compared to placebo in patients with extrapancreatic NETs (30% of whom had bronchial NETs). The PFS hazard ratio in the bronchial subgroup was 0.50 (95% CI 0.28–0.88), similar to the hazard ratio for the overall cohort [49]. Everolimus can result in dyslipidemia and worsening of glycemic control; pneumonitis is a serious but uncommon side effect, and patients on everolimus with persistent cough or dyspnea should undergo high-resolution CT of the chest. Cabozantinib is a recently investigated multitarget tyrosine kinase inhibitor, targeting MET and VEGFR2. The placebo-controlled CABINET trial showed that cabozantinib prolonged PFS in extensively pretreated patients with both pancreatic and extrapancreatic NETs [50]. Bronchial and thymic NETs comprised 24% of the cohort; the hazard ratio for this small subgroup was 0.17 (95% CI 0.07–0.42) in line with the overall findings. 

Response rates with both these agents are modest, being 5% for cabozantinib in extrapancreatic NETs and 1% (1/63) for everolimus in lung NETs. Surufatinib, an antiangiogenic TKI, showed PFS improvement in a phase III trial in extrapancreatic NETs, 12% of which were of lung origin [51]. However, surufatinib is not easily accessible outside China.

#### 4.2.3. Chemotherapy

Chemotherapy is standard-of-care treatment in advanced high-grade lung neuroendocrine carcinomas (for both large-cell and small-cell NECs) but is associated with less impressive response rates in the lower-grade setting. This may be related to the slower biology of TCs/ACs. Temozolomide chemotherapy, either as monotherapy or in combination with capecitabine (CAPTEM), has shown some evidence of activity in retrospective series [52,53]. The recent MGMT-NET trial included 38 patients with thoracic NET, randomizing patients to alkylating agent-based versus oxaliplatin-based chemotherapy. Patients with deficient MGMT status had greater ORR and PFS with alkylating-based therapy; those with proficient MGMT had a higher response rate with oxaliplatin-based therapy, but PFS was not significantly different [54]. Platinum-containing doublets may be used for rapidly progressive atypical carcinoids or supracarcinoids. Prospective data are required to guide optimal sequencing of these therapies.

#### 4.2.4. Immunotherapy

Immunotherapy has a limited role outside clinical trials in the management of typical and atypical carcinoids. Whilst there is significant evidence to support the use of PD-1/L1 inhibitors such as atezolizumab and durvalumab in the management of extensive-stage small-cell lung cancer, TC and ACs have a lower tumor mutational burden [55] and are therefore less amenable to immunotherapeutic approaches in general. Trials of combination CTLA-4/PD-1 blockade have shown modest results for both durvalumab/tremelimumab and ipilimumab/nivolumab [56,57]. Whilst the latter of these trials, a subgroup analysis of the CA209-538 trial, demonstrated response in 3 of 11 patients with bronchial NETs, the small numbers mean that firm conclusions cannot be drawn. There are data for the use of interferon in refractory cases, but it is not often used in practice due to its significant side effect profile. Multi-omic subtyping selecting an immune-active subset characterized by high DLL3 expression and heavy T-cell infiltration may suggest a potential biomarker-selected niche for immune checkpoint inhibition or other immunotherapies in lung carcinoids. Future trials focused on evaluating rational combination strategies, such as pairing immunotherapy with anti-angiogenic TKIs or PRRT, are eagerly awaited.

#### 4.2.5. Peptide Receptor Radionuclide Therapy (PRRT)

Peptide receptor radionuclide therapy (PRRT), most often with ^177^Lu-DOTA-octreotate, has been used in Europe for several decades for NET treatment and is increasingly used in North America. Whilst registration trials of PRRT predominantly included patients with GEPNETs, there are significant retrospective data attesting to the clinical efficacy of PRRT for bronchial NETs [58,59]. PRRT is injected intravenously and internalized systemically through somatostatin receptors (particularly subtype 2), resulting in beta irradiation of tumor deposits. PRRT eligibility should be defined by objective SSTR expression criteria (Krenning Grade ≥ 3 or tumor SUV_max_ > liver SUV_mean_) rather than absolute avidity across all sites, allowing for nuanced management of heterogeneous or discordant disease through targeted biopsy, localized therapy of non-avid lesions, or alternative systemic strategies. There are ongoing phase III trials investigating PRRT compared to everolimus for SSTR-avid lung NETs (e.g., LEVEL, NCT05918302), and the results will help clinicians determine optimal sequencing of these therapies. Factors favoring PRRT may include high, homogeneous SSTR avidity, bulky symptomatic disease, and rapid progression on somatostatin analogs, whereas everolimus may be preferred for its oral convenience, avoidance of nuclear medicine logistics, or in cases with low or heterogeneous SSTR expression. There is some emerging evidence about the combination of PRRT with local therapies for patients who have the majority of lesions DOTATATE-avid and 1–3 lesions non-avid. Whilst alpha-emitting PRRT therapies are an avenue of active investigation, further data are required prior to their adoption into mainstream clinical practice [60,61]. 

#### 4.2.6. Combination Approaches

Combinations of the above therapies are starting to be investigated in clinical trials. For instance, the combination of lanreotide (120 mg/4 weeks) and temozolomide (250 mg/d) in the phase II ATLANT study [62] demonstrated a median PFS of 9 months in 40 patients with progressive thoracic NETs and may be investigated in a larger study.

#### 4.2.7. Locoregional Therapies

Locoregional management of oligometastatic or liver-dominant advanced pulmonary carcinoids encompasses surgical resection, thermal ablation, stereotactic radiotherapy, liver-directed therapies (embolization or radioembolization), and palliative radiotherapy, alongside endobronchial recanalization for obstructive central lesions. Incorporating these targeted strategies alongside systemic therapies ensures a comprehensive, multidisciplinary approach to symptom control, local disease management, and tumor cytoreduction.

#### 4.2.8. Treatment Sequencing

Treatment sequencing in advanced pulmonary carcinoids should be guided by a framework evaluating functional status, SSTR and FDG avidity, tumor burden, and pace of progression rather than a standard linear approach. First-line therapy transitions from somatostatin analogs in indolent, SSTR-avid disease to targeted therapies (everolimus), PRRT, or cytotoxic regimens should be based on disease kinetics and regional drug access. Novel therapeutic approaches for advanced pulmonary carcinoids are shifting toward targeted, biomarker-driven strategies, including next-generation PRRT with alpha-emitters and multi-kinase inhibitors; however, these remain investigational in clinical trials.

**Table 1:** Summary of key clinical trials evaluating systemic therapies.

Trial	Drug	Phase/Design	Population/Thoracic Subgroup	Primary Endpoint	Key Outcomes	Thoracic Subgroup Findings
SPINET [48]	Lanreotide	Randomized phase III	Advanced bronchopulmonary NETs (n = 77; terminated early due to slow accrual)	PFS	Median PFS: 16.6 mo (lanreotide) vs. 13.6 mo (placebo)	TC median PFS: 21.9 mo; AC median PFS: 13.8 mo
RADIANT-4 [49]	Everolimus	Randomized phase III	Advanced non-functional extrapancreatic NETs (n = 302; 90/302 [30%] lung NETs)	PFS	Overall median PFS: 11.0 mo vs. 3.9 mo (HR 0.48, 95% CI 0.35–0.67, *p* < 0.00001)	Lung subgroup HR 0.50 (95% CI: 0.28–0.88); DCR 81%
CABINET [50]	Cabozantinib	Randomized phase III	Pretreated advanced extrapancreatic NETs (n = 203; 49/203 [24%] thoracic/thymic)	PFS	Extrapancreatic median PFS: 8.4 mo vs. 3.9 mo (*p* < 0.001)	Extrapancreatic subgroup HR 0.38 (95% CI: 0.25–0.59; *p* < 0.001)
SANET-ep [51]	Surufantinib	Randomized phase III	Advanced extrapancreatic NETs (n = 198; 23/198 [12%] lung origin)	PFS	Median PFS: 9.2 mo vs. 3.8 mo (*p* < 0.0001)	Extrapancreatic (lung, stomach, liver, vermiform appendix, colon, rectum) cohort HR 0.33 (95% CI: 0.2–0.503)
MGMT-NET [54]	Temozolomide-based vs. oxaliplatin-based	Randomized phase II	Advanced NETs (n = 102; 38/102 [37%] thoracic NETs) randomized to alkylating vs. oxaliplatin-based chemotherapy by MGMT status	ORR at 3 mo	MGMT-deficient: higher ORR and PFS with alkylating regimen	MGMT-proficient: higher ORR with oxaliplatin-based therapy; PFS not significantly different
ATLANT [62]	Lanreotide plus temozolomide	Single-arm phase II	Progressive thoracic NETs (n = 40)	DCR rate at 9 mo	DCR at 9 mo was 35%; median PFS 37 weeks	Promising efficacy

## 5. Future Directions

Despite significant advances in the molecular characterization of pulmonary carcinoid tumors, several critical gaps remain in our understanding of their underlying biology. Recent genomic, epigenomic, and transcriptomic studies have revealed substantial heterogeneity beyond the traditional typical–atypical dichotomy, including biologically distinct subgroups and aggressive supra-carcinoid subsets [15,19,20,28,29]. However, the precise mechanisms linking molecular subtypes to clinical behavior and treatment response remain incompletely defined. In particular, how transcriptional programs, proliferative activity, functional imaging phenotype, and the tumor immune microenvironment interact to drive disease progression and therapeutic vulnerability remains a major area for future investigation [15,19,36,37]. 

Looking ahead, clinical management is likely to shift from a static, morphology-driven framework toward a dynamic, biology-driven model. Treatment selection should increasingly integrate histology, Ki-67, functional imaging, and molecular or immunohistochemical markers such as OTP/CD44, ASCL1/OTP expression patterns, TERT alterations, MGMT status, SSTR2A expression, and DLL3 expression [2,13,14,15,16,21,25]. In this framework, SSTR ^68^Ga PET positivity may support the use of somatostatin analogs or PRRT, whereas FDG-avid, SSTR-negative, or spatially discordant disease may indicate more aggressive biology and the need for alternative systemic approaches or repeat biopsy [35,36,37,57,58]. 

Future trials should therefore be designed around clinically meaningful biological subsets rather than broad extrapolation from gastroenteropancreatic NETs. For example, patients with SSTR-positive progressive lung carcinoids may be better suited to PRRT-based strategies, including ongoing randomized studies comparing PRRT with everolimus, whereas patients with rapidly progressive disease, higher Ki-67, or suspected molecular evolution may require chemotherapy-focused or biomarker-adaptive approaches [57,58,63]. The role of temozolomide-based therapy should also be clarified in relation to MGMT deficiency or methylation, particularly in light of recent prospective NET data and the phase II ATLANT study of lanreotide autogel plus temozolomide in progressive thoracic neuroendocrine tumors [51,52,54,62]. Novel immune-engaging or DLL3-directed strategies should be considered exploratory and tested prospectively in carefully selected pulmonary carcinoid subsets rather than assumed to apply broadly [15,17,25]. 

Liquid biopsy and ctDNA profiling may enable non-invasive, real-time monitoring of molecular evolution in lung carcinoids, offering early detection of high-grade transformation and aggressive subsets like supra-carcinoids. Incorporating serial ctDNA tracking into future trial designs will be key to optimizing personalized treatment sequencing.

Given the rarity and biological heterogeneity of pulmonary carcinoids, conventional one-size-fits-all trial designs are unlikely to be sufficient. Umbrella, basket, and adaptive trial designs that incorporate histology, functional imaging, molecular subtype, and dynamic markers of progression may be more appropriate for identifying biologically meaningful treatment effects. While emerging molecular classifications and biomarker-driven strategies hold significant promise, current management of advanced pulmonary carcinoids relies heavily on extrapolated data from mixed neuroendocrine cohorts, underscoring the urgent need for prospective thoracic-dedicated trials to validate these paradigms in clinical practice. Ultimately, progress in this disease will depend not only on new drugs but also on aligning therapeutic sequencing with tumor biology, imaging phenotype, and clinical tempo.

## Data Availability

No new data were created or analyzed in this study. Data sharing is not applicable to this article.
